# Prevention approaches in a preclinical canine model of Alzheimer’s disease: benefits and challenges

**DOI:** 10.3389/fphar.2014.00047

**Published:** 2014-03-21

**Authors:** Paulina R. Davis, Elizabeth Head

**Affiliations:** ^1^Sanders-Brown Center on Aging, University of KentuckyLexington, KY, USA; ^2^Department of Molecular and Biomedical Pharmacology, University of KentuckyLexington, KY, USA

**Keywords:** antioxidant diet, atorvastatin, behavioral enrichment, beta-amyloid, combination treatment, dog, immunotherapy, statin

## Abstract

Aged dogs spontaneously develop many features of human aging and Alzheimer’s disease (AD) including cognitive decline and neuropathology. In this review, we discuss age-dependent learning tasks, memory tasks, and functional measures that can be used in aged dogs for sensitive treatment outcome measures. Neuropathology that is linked to cognitive decline is described along with examples of treatment studies that show reduced neuropathology in aging dogs (dietary manipulations, behavioral enrichment, immunotherapy, and statins). Studies in canine show that multi-targeted approaches may be more beneficial than single pathway manipulations (e.g., antioxidants combined with behavioral enrichment). Aging canine studies show good predictive validity for human clinical trials outcomes (e.g., immunotherapy) and several interventions tested in dogs strongly support a prevention approach (e.g., immunotherapy and statins). Further, dogs are ideally suited for prevention studies as they the age because onset of cognitive decline and neuropathology strongly support longitudinal interventions that can be completed within a 3–5 year period. Disadvantages to using the canine model are that they lengthy, use labor-intensive comprehensive cognitive testing, and involve costly housing (almost as high as that of non-human primates). However, overall, using the dog as a preclinical model for testing preventive approaches for AD may complement work in rodents and non-human primates.

## INTRODUCTION

Alzheimer’s disease (AD) is a progressive dementia associated with the accumulation of beta-amyloid (Aβ) plaques and neurofibrillary tangles (NFT; McKhann et al., 1984; Mirra, 1997). Currently five drugs are approved for use by the FDA to manage the symptoms of AD, although none target disease pathways and all may provide only symptomatic relief. These drugs include donepezil, rivastigmine, tacrine, galantamine, and memantine (Aisen et al., 2012). However, these five approved drugs target only two pathways, one involving acetylcholinesterase inhibition and the second is an NMDA receptor antagonist. Thus, preclinical studies are critical for developing and testing new disease-modifying interventions that can be taken to clinical trials in patients with AD. Typically, studies in rodents are the earliest steps in this process to screen drugs that target AD pathways with most preclinical studies of AD interventions using transgenic mouse models of AD. Subsequently, safety studies in humans are followed by a clinical trial in AD patients. Many of the AD clinical trials currently underway target different pathogenic pathways active in the disease^1^.

Several clinical trials are targeting the reduction of Aβ. The rationale stems from predictions based on the amyloid hypothesis, originally proposed by Hardy and Higgins (1992) and updated by Hardy (2006) suggesting that Aβ is a critical causative factor in the disease. Thus, the focus of several clinical trials has been to either reduce production (secretase inhibition) or increase clearance (immunotherapy) of Aβ. Unfortunately, most of these promising new approaches have failed in clinical trials (for review, see Mullane and Williams, 2013). Possible reasons for failure include but are not limited to: (1) the targets are not critical for AD pathogenesis and dementia, (2) the single pathway reductionist approach may be insufficient, (3) the treatment is too late (suggesting prevention studies) or, (4) the preclinical animal model was not a predictor of human clinical trials outcomes. In this review we first discuss the canine model of human aging and AD, how dogs are well suited for prevention studies based on established sensitive cognitive tasks and brain pathology measures, and then outcomes of preclinical studies with both single and multiple targets that may predict human clinical trial outcomes.

## THE CANINE MODEL OF HUMAN AGING AND ALZHEIMER’S DISEASE

Some of the most commonly studied animal models of human brain aging are rodents and non-human primates (Gallagher and Rapp, 1997). Other animals, including wolves, bears, cats, and dogs, naturally develop human-like neuropathology (Head et al., 2001). Of these animals, cats and dogs tend to have similar living environments to humans (Head et al., 2001). Canines, however, show cognitive decline with age and develop most aspects of neuropathology seen in aged human brain including AD patients (Cummings et al., 1996b; Cotman and Head, 2008). Such neuropathology includes Aβ pathology, reduced brain volume, neuronal loss, and impaired neurogenesis (Head, 2001; Cotman and Head, 2008). In addition to the similar cognitive decline and accumulation of neuropathological hallmarks to humans with AD, drugs exhibit similar pharmacokinetics when administered to dogs or humans [for example statins – (Gerson et al., 1989; Alberts, 1990)], making them an appropriate model for translational studies on therapeutic drugs. Not only are dogs easy to handle due to their long history of domestication, but pet dogs also share similar living conditions and diets to humans (Cummings et al., 1996b; Parker et al., 2004; Axelsson et al., 2013). Canines are highly motivated by food reward when conducting cognitive tests, which makes them cooperative research subjects by reducing or eliminating deprivation protocols for motivation. Thus, this cooperativeness eliminates many physiological stressors that can affect cognitive testing results present in other animal models such as rodents that require food deprivation or cold water for motivation (Blizard et al., 2003). The similar cognitive decline and accumulation of neuropathology to humans makes the canine model of aging useful for translational research on neurodegenerative diseases, especially AD.

## COGNITIVE OUTCOME MEASURES FOR PREVENTION STUDIES IN AGING DOGS

We describe several measures of cognition that are age-sensitive and treatment-sensitive in dogs that can be used as intervention outcome measures to assess different cognitive abilities with analogous tasks in non-human primates and in humans (**Table 1**). Much like humans, the aging canine shows cognitive decline with various cognitive domains and cortical pathways being differentially affected (Milgram et al., 1994). Dogs show cognitive deficits due to age in tests measuring complex learning, executive function, spatial learning and attention, and memory (Milgram et al., 1994, 2002b; Head et al., 1998a; Cotman et al., 2002; Tapp et al., 2003a,b, 2004b; Christie et al., 2005; Siwak et al., 2005; Studzinski et al., 2006). In addition to cognitive domain variability, individual dogs also show variability in cognitive function as seen in humans (Adams et al., 2000). This variability becomes most apparent in old canines, and using spatial learning and memory tasks, we are able to distinguish three groups of variability: (1) successful agers, (2) impaired dogs whose scores fell two SD above the mean of the young animals, and (3) severely impaired dogs who failed to learn the task (Head et al., 2001). The availability of age-matched animals with and without cognitive deficits allowed researchers to determine which types of neuropathology contribute to individual cognitive impairments in these animals (e.g., Head et al., 1998a).

**Table 1:** Cognitive domains assessed in dog aging and comparison with non-human primate tasks and analogous tasks used in human neuropsychological testing.

Cognitive domain	Dog task	Localization in dog brain	Non-human primate tasks	Examples of human neuropsychological tasks^**^
Learning	Visual discrimination learning	Medial temporal lobe/parietal lobe^*^	Visual discrimination learning (Rapp, 1990; Lai et al., 1995)	Digit copy, rotary pursuit, face discrimination (Cronin-Golomb, 2001), object discrimination (Freedman and Oscar-Berman, 1989; Boutet et al., 2007)
Memory	Spatial delayed non-match to sample acquisition	Dorsolateral prefrontal cortex (Christie et al., 2005)	Delayed response task (Arnsten and Goldman-Rakic, 1985; Walker et al., 1988)	Delayed recognition and recall, digit span (Lezak et al., 2004)
	Spatial delayed non-match to sample memory	Hippocampus (Kowalska, 1995)	Delayed response task (Arnsten and Goldman-Rakic, 1985; Walker et al., 1988)	
Executive function	Visual reversal learning	Prefrontal cortex/medial temporal lobe (Warren, 1964)	Visual reversal learning (Rapp, 1990; Lai et al., 1995)	Card or object sorting tasks, set shifting, response inhibition (Kramer and Quitania, 2007)
	Oddity discrimination	Prefrontal cortex/medial temporal lobe^*^	N/A	
Visuospatial function	Landmark discrimination	Prefrontal cortex/parietal cortex^*^	Landmark discrimination (Pohl, 1973)	Visual construction, block design, spatial learning (Freedman and Oscar-Berman, 1989; Boutet et al., 2007)
	Egocentric spatial learning	Hippocampus/medial temporal lobe^*^*	Spatial learning (Lai et al., 1995)	

* Proposed localization – not confirmed in lesion studies in dogs.

** Neuropsychological tasks for humans that assess function in similar cognitive domains.

*Modified from *Table 1* in Martin et al. (2011b).
*

Several tasks, similar to those used for testing cognition in non-human primates, have been developed to measure cognitive decline in the aging canine (Milgram et al., 1994, 1999, 2002a; **Table 1**). Such tasks include landmark discrimination, oddity discrimination, object, size and black/white discrimination and reversal tasks, and a spatial memory task. In our laboratory studies using these cognitive tasks, all testing occurs in a modified Wisconsin General Testing Apparatus such that the motor and sensory demands are consistent across tasks (Milgram et al., 1994). For each task, 10–12 trials are given per day and dogs are tested daily until a predetermined criterion level of performance is reached; total error scores are added up across days to provide a measure of learning and/or memory for each animal. These tasks are described in more detail below to illustrate how a test battery can be developed to measure the function of several brain circuits that may be differentially affected by age and/or treatment in aging dogs.

The *landmark discrimination task*, which measures visuospatial function and allocentric learning, involves presenting dogs with two identical objects, one of which is adjacent to a third object that serves as a landmark (Milgram et al., 1999). Animals are required to recognize that the landmark is an indicator of which object covers the food reward, and selection of the object closest to this landmark by the animal is considered a correct response. The task is made successively more difficult by placing the landmark further away from the object covering the reward. Previous work shows that aged dogs are impaired on the landmark task and show age decrements in their ability to determine how close the landmark is to the correct object (Milgram et al., 1999, 2002a).

The *oddity discrimination task* measures complex learning, as well as prefrontal cortex function (Cotman et al., 2002). Aged dogs show deficits in oddity discrimination learning (Cotman et al., 2002; Milgram et al., 2002b). In this task, dogs are presented with three objects simultaneously, two of which are identical and a third that is unique. A correct response is indicated when the dog chooses the unique object, resulting in a reward. To prevent a floor effect and detect progressive age decline, the oddity aspect of this task is made successively more difficult. Animals progress through four sets of three objects and each subsequent set contains a unique object, which is more difficult to distinguish from others than the previous set (Milgram et al., 2002b). Interestingly, young dogs can solve this problem by using the strategy of selecting the novel object for each successive set of objects such that error scores plateau; in contrast, aged dogs do not learn a strategy but re-learn each set of objects as a new problem (Cotman et al., 2002; Milgram et al., 2002b).

Tests of *object, size and black/white discrimination* are administered to measure associative learning ability. Object discrimination involves presenting dogs with two different objects simultaneously with one of the two objects consistently rewarded. Dogs must learn to select the same object each presentation with the left/right position being randomly determined. Similarly, the size discrimination objects differ in size (small/large) and the black/white discrimination task objects differ only in color (black/white; Milgram et al., 2005). Object, size and black/white discrimination are also progressively more difficult for animals to solve given the similarities in the objects increasing. Thus, these three tasks in combination can serve as different test versions (much like in clinical studies in people) to assess longitudinal changes in learning while reducing practice effects (Milgram et al., 2005).

Executive function can be evaluated immediately after discrimination learning has been completed by using the *object, size or black/white reversal* objects. The reversal tasks differ from the original discrimination task in that the positive and negative objects for reward contingencies are reversed after animals have learned the initial discrimination (Milgram et al., 2004, 2005). Reversing the reward contingencies can show perseverative behaviors (persistent choice of previously correct object), which are frontal cortex dependent (Warren, 1964). A subset of the discrimination learning tasks and all reversal learning tasks are age-dependent, with reversal learning being consistently more impaired with age (Milgram et al., 1994, 2004, 2005; Tapp et al., 2004b; Siwak et al., 2005).

Memory also declines with age in dogs. The most useful age-sensitive task we have used is a *spatial memory task*, in which dogs are required to recognize the location of a sample stimulus and then respond to a different location during the test trial. We refer to this as a delayed non-match to position task (DNMP) and it involves showing animals a single object covering a food reward either on the left or right food well. After animals move the object and obtain the reward, the object is withdrawn from sight for a predetermined delay period (e.g., 10 s). Subsequently animals are given two identical objects to choose from; one is the same object in the same position as before and one is in a novel position. The correct response is to select the object covering the novel location. Results published in 1995, Head et al. (1995) suggested that the task was age-sensitive. We subsequently developed a three-choice visuospatial working memory task that allows determination of the differential age-dependent strategies (e.g., cognitive or stimulus-dependent strategies) dogs use in solving the problem (Chan et al., 2002). In this task, rather than just the left and right food wells are used but a center well is also included to make the task more difficult. Further, this task shows minimal practice effects in longitudinal studies (Head et al., 2008). We identified the time course of the development of cognitive decline and found that deterioration in spatial ability occurs early in the aging process, between 6 and 7 years of age in dogs (Studzinski et al., 2006).

## BEHAVIORAL/FUNCTIONAL OUTCOME MEASURES FOR PREVENTION STUDIES IN DOGS

In addition to cognitive outcome measures, researchers and veterinarians are interested in measuring functional outcomes. Further, laboratory-based cognitive testing as described above is labor intensive and requires many months to years to obtain data. An *open field test* can be used to observe the behavioral patterns of animals in an empty room for 10 min. During this task, movement, sniffing, urinating, grooming, rearing, jumping, vocalization, and inactivity are noted (Head and Milgram, 1992; Siwak et al., 2000, 2001). *Self-recognition* can be evaluated through the mirror test, originally developed for primates (Gallup, 1968; de Veer et al., 2003), by observing the reaction of each animal with a mirror and their reflection. *Exploratory behavior* of canines can be assessed through a curiosity test in which animals are presented with various novel play objects. During their time with the objects, the amount of time the dogs spend in physical contact with or sitting next to the objects is recorded as well as their frequency of sniffing the objects (Siwak et al., 2001). Social responsiveness of dogs can be gaged through a few different tasks: a human interaction test, silhouette test, and the model dog test. A *human interaction* test is performed by the presence of a person in the middle of the room and recording the reaction of the dog to that person by measuring the time the dog is in physical contact with the person, time sitting or standing beside the person, and frequency sniffing the person (Head et al., 1997). The *silhouette test *records the animals frequency of sniffing the front and rear regions of a cardboard silhouette of a dog posted onto a wall (Fox and Weisman, 1970). The model dog test also records the sniffing frequency of the dogs, but this time in response to the presence of a life size model dog in the center of a room (Siwak et al., 2001).

Behavioral patterns in these tasks show age effects as well as differential effects based on the presence of intact/impaired cognition. Siwak et al. (2001) characterized the behavioral profiles of young (2–4 years), aged (9–15 years) cognitively impaired, and aged non-impaired beagles. Young dogs tend to show greater responsiveness to changes in environments such as the addition of novel objects and a person. They also showed greater social responsiveness spending the most time next to or sniffing a person, silhouette, and model dog. Aged unimpaired dogs were still responsive to alterations in environment, but to a lesser degree than the young animals. Additionally, aged unimpaired dogs spent the least amount of time reacting to the mirror during the self-recognition task. Unlike either the young or aged unimpaired canines, the aged impaired canines were unresponsive to all stimuli presented to the environment and randomly moved about the room in pacing/aimless behavior. However, the aged impaired dogs did spend the most time interacting with the mirror in the self-recognition test (Siwak et al., 2001).

Measures of canine function can also be assessed in a clinical setting (Landsberg and Ruehl, 1997; Landsberg and Araujo, 2005; Landsberg et al., 2012). Clinical measures have been developed consisting of pet *dog owner based evaluation* of dog behavioral changes (Colle et al., 2000; Pugliese et al., 2006a, 2007; Bosch et al., 2012, 2013; Landsberg et al., 2012) similar to those used in human clinical evaluations, such as the mini mental state exam (MMSE). Although there are different versions of these questionnaires, all appear to be sensitive to the presence of canine cognitive dysfunction (Landsberg et al., 2012). The evaluation consists of items such as walking, posture/emotion of expression, elimination behavior, life rhythm, play behavior, exploratory behavior, learned specific behavior, adaptive capabilities, and interactions with other animals or with owners. The items of individual questionnaires can be used to derive scores that distinguish between normally and pathologically aging dogs. Adult and older dogs generally score worse with these types of evaluation tools, and old dogs show individual variability in terms of the amount of cognitive dysfunction reported (Bosch et al., 2012).

## DOG NEUROPATHOLOGY AND OUTCOME MEASURES FOR PREVENTION STUDIES

Just as canines can exhibit cognitive decline with age similar to aging humans and patients with AD, several human-type neuropathologies have been reported in dogs (Cotman and Head, 2008). In particular, the canine model has long been suggested as an excellent model of Aβ pathogenesis (Wisniewski et al., 1990). Several changes observed in the aged canine brain are associated with cognition and are discussed below.

Individuals with AD show significant cortical and hippocampal atrophy relative to non-demented age matched controls (Alavi et al., 1993; Raz et al., 1998) and losses *in brain volume* correlate with cognitive decline (Ezekiel et al., 2004; Du et al., 2005). Similar events are seen in aged canines. On cross sectional MR imaging, aging canines show increased cortical atrophy and ventricular widening (Su et al., 1998; Gonzalez-Soriano et al., 2001; Kimotsuki et al., 2005). Ventricular widening over time was observed by MRI in a 3-year longitudinal study (Su et al., 2005). Canine cortical atrophy occurs earliest in the prefrontal cortex and later with age in the hippocampus (Tapp et al., 2004a). As with humans, the more extensive the cortical/hippocampal atrophy seen in aged canines the more pronounced the cognitive deficits (Tapp et al., 2004a; Rofina et al., 2006).

*Neuronal loss* occurs in human brain aging and could explain the brain volume losses seen in brain imaging (West, 1993; Simic et al., 1997). With normal brain aging, neuronal loss is only seen in the hilus (West, 1993; West et al., 1994), while neuronal loss is much more widespread in individuals with AD (Bobinski et al., 1997; West et al., 2000). Individuals with AD experience neuronal loss in the CA1, CA2, CA4, and subiculum of the hippocampus (Bobinski et al., 1997; West et al., 2000; Price et al., 2001). In aged beagles, the hilus of the dentate gyrus showed fewer neurons compared to younger dogs (Siwak-Tapp et al., 2008). Beagles with fewer neurons in the hilus made significantly more errors when performing the size discrimination task (Siwak-Tapp et al., 2008). Similarly, Pugliese et al. (2007) found that a loss of Purkinje cells in canines correlated with data acquired by questionnaires quantifying behavioral deficits. However, neuronal loss may not account for all of the brain atrophy observed by MR as the loss of neuronal dendritic spines occurs with AD (Knobloch and Mansuy, 2008; Overk and Masliah, 2014) but to our knowledge, there are currently no studies published evaluating similar changes with age in dogs.

While selective neuronal loss may occur with aging, the brain is also able to produce new neurons. The hippocampus, for example, grows new neurons in the subgranular layer (Eriksson et al., 1998). *Neurogenesis* has been explored in aged beagles using BrdU and doublecortin protein staining methods. Siwak-Tapp et al. (2007) measured neurogenesis in aged beagles using BrdU and found that animals over the age of 13 experienced a significant loss of neurogenesis. Fewer newer BrdU positive neurons was associated with poorer cognitive function in learning and memory and learning ability (Siwak-Tapp et al., 2007).

Neuronal dysfunction could result in abnormal production of critical *neurotransmitters* in the brain. Thus, one potential target for therapeutics in AD is to manipulate or restore decreased neurotransmitter levels. Some drugs targeting neurotransmitters are already available as treatments for AD; however, as mentioned earlier; these drugs at best provide only symptomatic relief. Neurotransmitter deficits have not been thoroughly explored in canines. In humans, decreases in specific neurotransmitter systems are associated with aging and AD (Meltzer et al., 1998; Ballard et al., 2005; Schliebs and Arendt, 2006; Rissman et al., 2007). Dogs with Aβ accumulation in the gyrus proreus possess fewer serotonergic neurons (Bernedo et al., 2009). A decrease in receptor binding of serotonin is seen with age in dogs over 8 years of age (Peremans et al., 2002). Animals with high levels of Aβ in the prefrontal cortex experience a loss of noradrenergic neurons in the locus ceruleus, which is also associated with cognitive dysfunction (Insua et al., 2010). Acetylcholinesterase density is reduced in granule cells of the cerebellum with age (Pugliese et al., 2007). Aged canines experience a loss of gamma-aminobutyric acid interneurons in the prefrontal cortex (Pugliese et al., 2004), as well as the CA1 and dentate gyrus of the hippocampus (Hwang et al., 2008b). Additionally, a loss of glutamic acid decarboxylase 67 neurons in CA1 of the hippocampus is seen in aged canines over 10 years of age (Hwang et al., 2008b). Thus, similar patterns of age-associated neurotransmitter system dysfunction appear in aging dogs and may be a suitable model system in which to develop or test novel neurotransmitter pathway-based interventions. The pathogenic mechanisms underlying neuronal dysfunction, neurotransmitter losses and death may include, e.g., the deposition of Aβ, cerebrovascular dysfunction, or oxidative damage.

*Beta-amyloid* (*A*β) is derived from a longer precursor protein, the amyloid precursor protein (APP). The APP sequence of *Canis familiaris* has 98% homology with human APP^2^ and an identical amino acid sequence (Selkoe et al., 1987; Johnstone et al., 1991). Additionally, dog Aβ peptides may undergo the same posttranslational modifications as in humans (Satou et al., 1997; Azizeh et al., 2000). These similarities make canines a viable aging model without the need for genetic modification or overexpression of mutant human proteins (Selkoe et al., 1987).

**FIGURE 1:**
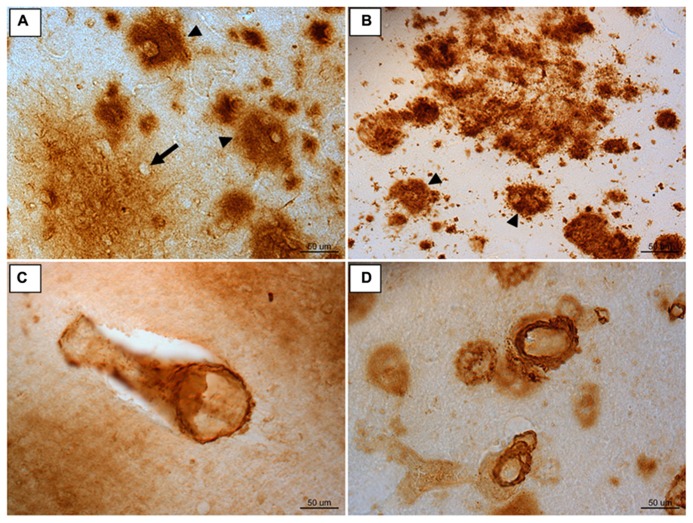
**Immunoreactivity for Aβ 1-42 in frontal cortex brain tissue of **(A)** an aged canine and **(B)** an aged human.** Compact Aβ deposits are similar in humans and canines (arrow head). The outline of an intact neuron enveloped by a diffuse plaque is visible (arrow). Aβ 1-40 immunoreactivity of cerebral amyloid angiopathy is similar in aged canine occipital cortex **(C)** and aged human occipital cortex **(D)**. Reproduced from Martin et al. (2011b).

The Aβ present in canines is ultrastructurally fibrillar and, though more compact deposits may form, it generally aggregates into diffuse plaques (Giaccone et al., 1990; Russell et al., 1992; Uchida et al., 1992; Cummings et al., 1993; Morys et al., 1994; Torp et al., 2000a,b). This type of Aβ deposition most resembles early AD pathology (Morris et al., 1996; Markesbery et al., 2006; Cotman and Head, 2008; **Figures 1A,B**). Since most AD therapeutics studied today are likely to have a greater affect if applied earlier in the disease progression, the early AD-like pathology canines produce makes them an attractive model for prevention studies (Martin et al., 2011b). As with cognitive decline, AD-like neuropathology has a region specific progression in both humans and canines (Wisniewski et al., 1970; Selkoe et al., 1987; Giaccone et al., 1990; Braak and Braak, 1991; Head et al., 2000; Thal et al., 2002). Though this progression in dogs is similar to that reported in humans, it is not identical. In canines, the accumulation of Aβ begins in the prefrontal cortex (approximately 8 years at age of onset) and continues to develop with increasing age to include other regions such as the temporal and occipital cortex (Russell et al., 1996; Head et al., 2000; Cotman and Head, 2008). The severity of neuropathology can vary between individual animals but can be linked to the extent of cognitive decline (Cummings et al., 1996a; Head et al., 1998b; Colle et al., 2000; Rofina et al., 2006). For instance, animals who perform worse in reversal learning tasks have greater Aβ pathology in the prefrontal cortex, while those deficient in size discrimination learning show higher amounts of Aβ in the entorhinal cortex (Cummings et al., 1996a; Head et al., 1998a; Pop et al., 2010b).

Aβ peptide can also be measured in the cerebrospinal fluid (CSF) of dogs (Sarasa et al., 2013). Measuring CSF Aβ as a ratio of Aβ 42/Aβ 40 is a good predictor of Aβ in the brain in dogs (Head et al., 2010). While brain Aβ increases with age, CSF Aβ decreases with age reflecting the hypothesis that Aβ migrates from the periphery and deposits in the brain with age and AD.

Aside from the fibrillar Aβ found in diffuse plaques in AD, a smaller, more soluble form of Aβ – oligomeric Aβ – is also seen in the aged dog brain. This more toxic form of Aβ affects synaptic function and can even be found in plaques (Walsh et al., 2002; Kayed et al., 2003; Selkoe, 2008). Higher levels of oligomers are present in canines and humans with increasing age and cognitive decline. The greater the cognitive deficit, the more prevalent oligomers are in the brain (Tomic et al., 2009; Pop et al., 2010a). Similar to fibrillar Aβ, oligomeric Aβ can be measured in CSF, where levels are inversely related to levels in the brain (Head et al., 2010).

Aβ can also aggregate in the cerebral blood vessel walls and cause *cerebrovascular pathology* (Prior et al., 1996; Attems, 2005; Herzig et al., 2006). This type of deposition is referred to as cerebral amyloid angiopathy (CAA; **Figures 1C,D**). Typically CAA is composed of the shorter Aβ 1-40 peptide (Wisniewski et al., 1996; Attems, 2005; Herzig et al., 2006). Both humans and canines exhibit CAA pathology, with a particular vulnerability in the occipital cortex (Attems et al., 2005). CAA impairs the blood brain barrier, vascular function, and can cause microhemorrhages (Uchida et al., 1990; Prior et al., 1996; Deane and Zlokovic, 2007). Because of these complications, CAA may contribute to cognitive decline in both humans (Ellis et al., 1996; Rensink et al., 2003; Nicoll et al., 2004; Attems, 2005) and canines (Giaccone et al., 1990; Uchida et al., 1990, 1991; Head, 2013). Much like humans, canines experience microhemorrhages with age (Uchida et al., 1991). These cerebral hemorrhages are present in both animals with and without CAA, but are more common in those with the blood vessel pathology (Uchida et al., 1991). Given the significant overlap of cerebrovascular pathology with AD, the spontaneous accumulation of CAA in dogs also offers as yet, an underappreciated model system to test the effects of cerebrovascular pathology on cognition and AD neuropathology.

Aβ deposition may lead to oxidative damage or *vice versa*, oxidative damage may lead to Aβ (Butterfield, 1997). Ultimately, *oxidative damage* accumulates with age and can lead to neuronal dysfunction and thus impact cognition (Butterfield et al., 2001). Oxidative damage occurs over time due to the overproduction of reactive oxygen species (ROS) produced primarily by mitochondria. When there is an overabundance of ROS, various mechanisms including production and release of endogenous antioxidants are in place to restore a homeostatic balance. However, ROS overproduction may exceed the levels or production rate of endogenous antioxidants and result in oxidative damage to proteins, lipids, and nucleotides. Oxidative damage can be measured by the amount of protein oxidation (carbonyl groups), 4-hydroxynonenal, lipofuscin, lipofuscin-like pigments, and malondialdehyde (lipid peroxidation). Further, 8-hydroxy-2′-deoxyguanosine (8OHdG) can be measured to detect DNA/RNA oxidation.

While oxidative damage occurs with normal aging, it is more pronounced in AD (Smith et al., 1991, 1996, 2000; Ames et al., 1993; Lovell et al., 1999; Montine et al., 2002; Pratico et al., 2002; Butterfield et al., 2007; Lovell and Markesbery, 2008), and similar patterns are seen in canines. In the canine model, there is evidence that ROS production is higher than in younger animals. In mitochondria isolated from aged canine brain, there is an increased production of ROS compared to mitochondria isolated from young animals (Head et al., 2009). Canines also experience an accumulation of carbonyl groups with age (Head et al., 2002; Skoumalova et al., 2003). Lipid peroxidation is exhibited in old dogs, measured by 4-hydroxynonenal (Papaioannou et al., 2001; Rofina et al., 2004, 2006; Hwang et al., 2008a), lipofuscin (Rofina et al., 2006), lipofuscin-like pigments (Papaioannou et al., 2001; Rofina et al., 2004), or malondialdehyde (Head et al., 2002). Increased 8OHdG in aged canines has also been reported (Rofina et al., 2006; Cotman and Head, 2008). In particular, increased protein oxidation and lipid peroxidation (lipofuscin-like pigment) correlates with cognitive decline in dogs (Skoumalova et al., 2003; Rofina et al., 2004, 2006). Given that canines exhibit age-associated oxidative damage in the brain that correlates with poorer cognition, these animals are suitable to study antioxidant treatment/prevention strategies.

One hallmark AD pathology canines do not produce is NFTs (Selkoe et al., 1987; Russell et al., 1992). While no research to date has observed NFTs in the canine brain, the increased phosphorylation seen at some sites of tau in AD cases also occurs in cognitively impaired canines (Kuroki et al., 1997; Wegiel et al., 1998; Papaioannou et al., 2001; Head et al., 2005; Pugliese et al., 2006b). This lack of NFT pathology could possibly be due to significant differences in the tau protein sequence between canines and humans^3^. However, an advantage to dogs not accumulating NFTs is that they serve as a model that is selective for Aβ pathology and ideally suited for testing interventions that target this toxic protein.

## TREATMENT STUDIES IN AGED DOGS AND PREDICTING HUMAN CLINICAL TRIALS

Several studies have tested therapeutic strategies using the canine model of aging and AD with both cognitive and neuropathological outcome measures (**Table 2**). Several of these involve dietary and/or environmental manipulations. One of the earliest studies to develop a treatment for cognitive dysfunction in aged dogs tested *an antioxidant-rich diet in combination with behavioral enrichment. *The behavioral enrichment included increased exercise, interaction with other dogs, and cognitive enrichment (Cotman et al., 2002; Milgram et al., 2002a, 2004, 2005). The diet included vitamins E and C, fruits and vegetables, lipoic acid and carnitine. Compared to control animals, those receiving an antioxidant-rich diet committed fewer errors during landmark acquisition and retention tasks (Milgram et al., 2002a) as well as oddity discrimination tasks (Cotman et al., 2002). Treatment with an antioxidant diet and behavioral enrichment resulted in improved performance during black and white object discrimination and reversal (Milgram et al., 2005). Pop et al. (2010b) found dogs provided with both behavioral enrichment and an antioxidant diet have an overall reduction in Aβ pathology across multiple regions of the brain. However, when looking at group treatment effects, only the antioxidant-treated animals had a significant reduction in Aβ plaque pathology. Additionally, the combination treatment approach of behavioral enrichment and an antioxidant-rich diet in aged canines was unable to reduce existing brain Aβ (Pop et al., 2010b). While plaque load was affected by the dual intervention, soluble and insoluble Aβ 1-40 was not affected, and only soluble levels of Aβ 1-42 were lowered specifically in the prefrontal cortex. A trend toward a significant decrease in oligomers specifically in the parietal cortex was observed in canines receiving the combined treatment (Pop et al., 2010b). Interestingly, the combination group also showed reduced oxidative damage (Opii et al., 2008) with the antioxidant diet group alone showing reduced mitochondrial dysfunction (Head et al., 2009). Further, the behavioral enrichment group, independent of the antioxidant diet treatment showed less neuron loss in the hippocampus (Siwak-Tapp et al., 2008) as well as improved levels of brain derived growth factor (Fahnestock et al., 2010).

**Table 2:** Cognitive outcomes of treatment studies in aging dogs^*^.

Treatment	Sample size and age	Landmark discrimination	Oddity discrimination	Size discrimination	Size reversal	Black white discrimination	Black/white reversal	Spatial memory	Questionnaire	Publication
Antioxidant diet	28 old (8–13 years)	Improved	Improved	Improved	Improved	Improved	Improved	Improved	Not assessed	Cotman et al. (2002), Milgram et al. (2004),^2005^
Behavioral enrichment		Not assessed	Not assessed	Improved	Improved	Improved	Improved	Improved	Not assessed	
Antioxidant diet + behavioral enrichment		Improved	Improved	Improved	Improved	Improved	Improved	Improved	Not assessed	
MCT dietary supplement	24 old (9–10 years)	Not improved	Impaired	Not assessed	Not assessed	Not assessed	Not assessed	Impaired	Not assessed	Pan et al. (2010)
Medical food cocktail	18 old (8–9 years)	Improved	Not improved	Not improved	Not improved	Not improved	Not improved	Not improved	Not assessed	Head et al. (2012)
Atorvastatin	10 old (9–13 years)	Not assessed	Not assessed	Not improved	Impaired	Not improved	Not improved	Not improved	Not assessed	Murphy et al. (2010)
Fibrillar Aβ1-42 immunotherapy	20 old (8–13 years)	Not improved	Not improved	Not improved	Maintained	Not improved	Maintained	Not improved	Not assessed	Head et al. (2008)
Fibrillar Aβ 1-40 and x-40 immunotherapy	12 old (11–18 years)	Not assessed	Not assessed	Not assessed	Not assessed	Not assessed	Not assessed	Not assessed	Improved	Bosch et al. (2013)

* Not an exhaustive list.

*Supplemental medium-chain TAG* (MCT) increases ketone levels in the brain, and these ketones can in turn be used as an alternative energy source. Pan et al. (2010) measured cognitive effects seen due to this supplement on the landmark discrimination, oddity discrimination, and two choice egocentric spatial learning tasks. Results indicated aged dogs given a diet with MCT supplementation performed better than those receiving a control diet in all tasks (Pan et al., 2010).

In contrast, fewer benefits on cognition were observed in a study using a *medical food cocktail* (Head et al., 2012). Dogs receiving a combination cocktail containing an extract of turmeric containing 95% curcuminoids, an extract of green tea containing 50% epigallocatechin gallate, N-acetyl cysteine, R-alpha lipoic acid and an extract of black pepper containing 95% piperine exhibited fewer errors compared to control animals during the landmark task indicating improved spatial attention. However, other areas of cognition were unaffected and brain Aβ remained unchanged (Head et al., 2012).

In 2008, a therapeutic approach that directly targeted Aβ reduction was explored in which aged beagles were actively immunized with fibrillar Aβ1-42 for 2 years (Immunized - IMM) based upon previous work in transgenic mouse models of AD (Schenk et al., 1999). This *immunotherapy* approach led to no improvement in cognitive function, but interestingly a long term maintenance of executive function was noted based on error scores from the size reversal learning task (Head et al., 2008). However, significant benefits to brain pathology were observed in the IMM dogs who showed significantly decreased Aβ plaque load in prefrontal, entorhinal, and occipital cortical regions, as well as reduced CAA (Head et al., 2008). While soluble and insoluble brain Aβ 1-40 and 42 significantly decreased in treated canines, there was no significant reduction in soluble oligomers. This study suggests that reducing or eliminating pre-existing Aβ in aging dogs is not sufficient to improve cognition.

Outcomes from the longitudinal dog vaccination study are similar to reports of the clinical trial in patients with AD where no differences between antibody responders and placebo groups on several cognitive and disability scales was observed. A small number of patients enrolled in the AN1792 study have come to autopsy and show Aβ plaque reduction without any effect on the extent of NFT or CAA (Nicoll et al., 2003; Ferrer et al., 2004; Masliah et al., 2005). Further, the frontal cortex showed the largest response to immunotherapy (Masliah et al., 2005), which is similar to our observations in the dog. The most recent autopsy study of eight patients that were in the AN1792 study further confirm reduced Aβ pathology in response to treatment, 5 years after the last injection (Holmes et al., 2008). However, reduction of brain Aβ did not slow disease progression and seven of eight patients had severe end stage dementia prior to death. (Gilman et al., 2005). Interestingly, a composite score of a neuropsychological test battery indicated “less worsening” of decline in antibody responders after 12 months and an improvement in the memory domain (Gilman et al., 2005).

Bosch et al. (2013) recently showed benefits of an active fibrillar Aβ_40_ and Aβ_x-40_ combination vaccine on cognition in aged housed beagles and pet dogs treated for 51 days. Over the course of treatment, cognitive evaluations by questionnaire were given at 31 days post treatment and at the end of treatment. Immunized animals showed a significant improvement in cognitive evaluation scores at both 31 and 51 days post treatment compared to pre-immunized scores (Bosch et al., 2013). Differences in the formulation, the outcome measures or the source of animals may explain the positive effects in the Bosch study compared with the previous beagle vaccine studies.

Several studies in the aged dog have tested the effects of drugs already approved for use in humans, with novel applications to brain aging. For example, several cross-sectional or case-control epidemiological studies revealed a striking link between *cholesterol-lowering drugs* (e.g., statins and others) and a 20–70% reduction in risk of developing AD (Jick et al., 2000; Wolozin et al., 2000, 2007; Hajjar et al., 2002; Rockwood et al., 2002; Rodriguez et al., 2002; Zamrini et al., 2004; Dufouil et al., 2005). Modest cognitive benefits have been reported in preliminary AD clinical trials with simvastatin (Simons et al., 2002) and atorvastatin (Sparks et al., 2005a,b, 2006a,b). In particular, AD patients with mild to moderate dementia who were treated with 80 mg/day atorvastatin had significantly improved scores on one measure of cognition Alzheimer’s Disease Assessment Scale-Cognitive subscale (ADAS-Cog) at 6 months of treatment, with smaller non-significant benefits at 12 months (Sparks et al., 2005b).

Statins may reduce the risk of incident AD through the prevention of Aβ production (Simons et al., 1998; Hartmann, 2001). In rodent models, treatment with inhibitors of 3-hydroxy-3-methylglutaryl coenzyme A (HMG-CoA) or statins reduces Aβ (Petanceska et al., 2002). However, rodents respond to statin treatment by massively upregulating HMG-CoA reductase levels (Fears et al., 1980; Alberts, 1990; Todd and Goa, 1990; Thelen et al., 2006). To compensate, long-term studies in rodent often employ physiologically excessive doses, making it difficult to translate the results of these studies into human trials. 

The dog model is particularly useful to study chronic statin treatment, given similarities with humans in terms of dose requirements, responsiveness, drug handling, and metabolism (Gerson et al., 1989; Alberts, 1990). For example, 12 dogs were treated with 80 mg/day of atorvastatin for 14.5 months (Murphy et al., 2010). Peripheral levels of cholesterol, low density lipoproteins, triglycerides and high density lipoproteins were reduced in treated dogs. Surprisingly, a transient impairment in reversal learning was observed, suggesting prefrontal dysfunction. Spatial memory remained unchanged up to over a year of treatment. The lack of cognitive benefits of treatment was also reflected by a lack of reduction in plasma, CSF, and brain Aβ. Interestingly, BACE1 protein level was decreased in the brains of atorvastatin-treated dogs. This intriguing outcome may suggest that statins might be more useful to prevent the production of Aβ through lowering BACE1 if started in animals in middle age, consistent with human studies indicating that middle-aged individuals using statins are protected from AD.

More recent work on the brain from statin-treated aged dogs suggests that additional benefits of atorvastatin include reducing oxidative damage and upregulating endogenous protective pathways. Thus, statins may have multiple benefits to the brain by affecting several pathways impaired by aging (Barone et al., 2011, 2012; Martin et al., 2011a; Butterfield et al., 2012). Aged dogs are a unique model that may provide novel insights and translational data to predict outcomes of statin use in human clinical trials.

## SUMMARY

Aged dogs capture many features of human aging and AD including cognitive decline and neuropathology. Canine studies show that multi-targeted approaches may be more beneficial than single pathway manipulations (e.g., antioxidants combined with behavioral enrichment vs. Aβ vaccine). Further, prevention studies could be accomplished in a 5-year period to test the effects of an intervention on the development of cognitive decline and neuropathology. Interestingly, an immunotherapy study in aged dogs illustrates the predictive validity of using this model system as aged dogs did not show cognitive improvements with an Aβ vaccine despite showing significant brain Aβ reductions, much like reports in the AD clinical trial. The canine model has numerous advantages as described above, however, systematic cognitive testing can be a lengthy and costly (given per diem rates) process and requires significant technical support. Still, the canine model should be considered an option since it is less involved and costly than a human clinical prevention study. Overall, using the dog as a preclinical model for testing preventive approaches for AD may be a useful step that complements work in rodents and non-human primates.

## Conflict of Interest Statement

The authors declare that the research was conducted in the absence of any commercial or financial relationships that could be construed as a potential conflict of interest.

## Footnotes

1 http://clinicaltrials.gov/ct2/results%3Fterm=alzheimer%27s%26pg=6

2 http://www.ensembl.org/Canis_familiaris/

3 http://www.ensembl.org/Canis_familiaris/
